# The Real DAPSI: A Real-World Retrospective Study on Assessing the Efficacy and Safety of a Fixed-Dose Combination of Dapagliflozin and Sitagliptin in the Indian Population

**DOI:** 10.7759/cureus.46767

**Published:** 2023-10-09

**Authors:** Rana Bhattacharjee, Madhukar Rai, Priyanka Joshi, Ashish Prasad, Ashish Birla

**Affiliations:** 1 Endocrinology, Diabetes and Metabolism, Institute of Post Graduate Medical Education & Research, Kolkata, IND; 2 Medicine, Heritage Institute of Medical Sciences, Varanasi, IND; 3 Medical Affairs, USV Private Limited, Mumbai, IND; 4 Scientific Services, USV Private Limited, Mumbai, IND; 5 Scientific Services, USV Private limited, Mumbai, IND

**Keywords:** real-world study, indian population, fixed-dose combination, sitagliptin, dapagliflozin, diabetes mellitus

## Abstract

Introduction

Type 2 diabetes mellitus (T2DM) is a chronic metabolic disorder affecting millions of individuals worldwide. Effective management of T2DM is crucial to prevent complications. Dapagliflozin and sitagliptin are oral anti-diabetic agents that have been shown to provide synergistic effects in controlling blood glucose levels. However, there is limited data on the efficacy and safety of the dapagliflozin-sitagliptin fixed-dose combination (FDC) in the Indian population. This study aimed to evaluate the real-world effectiveness and safety of the dapagliflozin-sitagliptin FDC in the Indian population.

Methods

This was a retrospective study conducted at healthcare centers in India. The study included patients with T2DM who were prescribed a FDC of dapagliflozin and sitagliptin. Data were collected from the medical health records of patients, including demographics, baseline glycated hemoglobin (HbA1c), blood glucose levels, BMI, blood pressure, and adverse events. The primary outcome was the change in HbA1c, postprandial plasma glucose (PPG), and fasting plasma glucose (FPG) from baseline to 12 weeks after treatment initiation.

Results

A total of 358 patients were included in the study, with a mean age of 56.2 years. The majority of the patients were male (68.2%), and the mean baseline HbA1c was 8.9 ± 0.87%. After 12 weeks of treatment with dapagliflozin and sitagliptin, there was a significant reduction in HbA1c levels from 8.9 to 7.2 (p <0.0001). There was also a significant reduction in fasting blood glucose levels from 178.8 to 124.0 (p <0.0001) and postprandial blood glucose levels from 273.9 to 176.0 (p <0.0001). There were no serious adverse events reported during the study period.

Conclusion

The FDC of dapagliflozin and sitagliptin is effective and safe in reducing blood glucose levels and BMI in the Indian population with T2DM. This real-world retrospective study provides valuable insights into the clinical effectiveness and safety of dapagliflozin-sitagliptin FDC in the Indian population. These findings highlight the potential benefits of this combination therapy in managing T2DM and pave the way for optimized treatment strategies and improved patient outcomes in the Indian healthcare landscape. Clinicians may consider dapagliflozin-sitagliptin FDC as a viable treatment option for T2DM patients.

## Introduction

Diabetes mellitus (DM) is a chronic metabolic disorder that is rapidly emerging as a global health challenge, affecting millions of individuals worldwide. India is currently the diabetes capital of the world, with an estimated 77 million people living with diabetes. Uncontrolled diabetes is associated with various long-term complications, such as cardiovascular disease, retinopathy, neuropathy, and nephropathy, which can significantly reduce the quality of life and lead to premature mortality. Therefore, effective management of diabetes is essential to prevent these complications and improve overall health outcomes [1,2].

Current guidelines recommend a multifaceted approach to type 2 DM (T2DM) management, including lifestyle modifications, oral anti-diabetic agents, and, if necessary, injectable therapies such as insulin or glucagon-like peptide-1 receptor agonists (GLP-1 RAs) [3]. Despite the availability of various treatment options, achieving and maintaining glycemic control remains a challenge for many patients, with a substantial proportion failing to achieve target glycated hemoglobin (HbA1c) levels [4]. Furthermore, the risk of cardiovascular and renal complications associated with T2DM necessitates therapeutic interventions that not only address glycemic control but also provide additional cardiovascular and renal benefits [5].

Dapagliflozin and sitagliptin are two anti-diabetic agents that work through different mechanisms to lower blood glucose levels. Dapagliflozin is a sodium-glucose cotransporter 2 (SGLT2) inhibitor that reduces renal glucose reabsorption, leading to increased urinary glucose excretion [3,6]. Sitagliptin, on the other hand, is a dipeptidyl peptidase-4 (DPP-4) inhibitor that increases the levels of active incretin hormones, which in turn stimulate insulin secretion and suppresses glucagon secretion. The combination of dapagliflozin and sitagliptin in a fixed-dose formulation has been shown to provide complementary glucose-lowering effects with a favorable safety profile in previous clinical trials [7].

Despite the promising results observed in clinical trials, real-world evidence is crucial to validate the effectiveness and safety of any therapeutic intervention. Real-world studies provide insights into treatment outcomes and adverse events in diverse patient populations under routine clinical practice, reflecting the true effectiveness of therapy in the general population. In the case of the fixed-dose combination (FDC) of dapagliflozin and sitagliptin, limited real-world evidence exists, particularly in the context of the Indian population [8].

The Indian population exhibits unique characteristics, such as a higher prevalence of insulin resistance, a distinct genetic profile, and disparities in healthcare access and quality. These unique characteristics of the Indian population make it imperative to evaluate the efficacy and safety of anti-diabetic agents in this population [9]. Furthermore, T2DM is often associated with comorbidities such as obesity, dyslipidemia, and hypertension, which further complicate management and may require multiple therapeutic interventions [10]. The FDC of dapagliflozin and sitagliptin offers a novel treatment option that addresses multiple aspects of T2DM management in a single tablet, potentially simplifying therapy, and improving patient adherence [11].

This real world study aims to bridge this knowledge gap by evaluating the real-world effectiveness and safety profile of the FDC of dapagliflozin and sitagliptin in Indian patients with T2DM. Moreover, the results of the real-world study for the FDC of dapagliflozin and Sitagliptin (real DAPSI) could have broader implications for the management of T2DM globally, given the growing prevalence of T2DM and the need for effective therapeutic options that minimize adverse events. The study's findings could provide insights into the real-world effectiveness and safety of combining SGLT2 inhibitors and DPP-4 inhibitors, potentially leading to improved glycemic control and reduced cardio-renal risk in patients with T2DM worldwide.

## Materials and methods

Study design

This study employed a retrospective observational design to investigate the real-world experience of the FDC of dapagliflozin and sitagliptin, with or without other anti-diabetic therapies, in patients with T2DM in India. The study was conducted across 50 sites in India and had a duration of 12 weeks. The primary endpoints of the study included the change in HbA1c after 12 weeks and the monthly changes in fasting plasma glucose (FPG) and postprandial plasma glucose (PPG) over a 12-week period. Secondary endpoints consisted of hypoglycemia incidence over 12 weeks, weight loss within 12 weeks, occurrences of urinary tract infections and genitourinary tract infections, as well as changes in blood pressure over a 12-week period.

Data collection and variables

For this study, medical records from various medical centers that treated patients with T2DM were selected. Approval was obtained from the treating physicians or medical practitioners, as well as the independent ethics committee (IEC) or institutional review board (IRB). Following approval from the site investigator and IEC/IRB, comprehensive patient-level information encompassing demographic, clinical, and laboratory variables were collected and entered into a digital case report form (CRF). Additionally, data regarding medication usage and comorbidities were recorded. The data regarding physicians' global evaluation of efficacy and tolerability physicians' global evaluation of efficacy and tolerability was collected through CRFs.

Inclusion and exclusion criteria

The inclusion and exclusion criteria were designed to ensure the enrollment of an appropriate patient population. In order to be included in the study, participants needed to be male or female patients over 18 years of age with HbA1c levels ranging between >7% and ≤10.5%. Furthermore, patients must have been receiving the FDC of dapagliflozin and sitagliptin for the treatment of T2DM, and their treating physician must have agreed to provide information regarding their treatment. Patients with incomplete data or those who, at the investigator's discretion, were deemed unsuitable for inclusion due to any underlying medical conditions, were excluded from the study.

Study population

The study population consisted of male or female patients aged 18 years or older who were diagnosed with T2DM and received treatment with the FDC of dapagliflozin and sitagliptin, as well as other anti-diabetic therapies, in accordance with the standard of care in a physician's practice. There was no limit on the number of patients that each site could select.

Statistical analysis

Descriptive statistics were employed to summarize baseline characteristics. Continuous variables were presented as mean and standard deviation, while categorical variables were expressed as frequencies and percentages. Appropriate statistical tests were used for data analysis. Data from all participating doctors were pooled for analysis, and adjustment for confounding variables was performed during the analysis.

Sample size

This study was considered a post-marketing study since the drug product had already been approved and was commercially available. A sample size of approximately 350 patients with T2DM was deemed sufficient to collect real-world experience, and a non-probability sampling method was utilized.

## Results

Patient demographics

A total of 358 patients were enrolled in this retrospective study. The patient demography and clinical characteristics are summarized in Table 1. The median duration of treatment with the FDC of dapagliflozin and sitagliptin was 7 +/- 4 months by the patients.

**Table 1:** Subject demographics and health status in overall population (N=358)

Parameter	Statistics	Overall (N=358)
Age (years)	N	358
	Mean	56.2
	SD	9.80
	Median	56
	Range: min, max	30.0, 88.0
	95% CI	(55.2,57.2)
Gender. n (%)	Male	244 (68.2)
	Female	114 (31.8)
BMI (kg/m^2^)	N	358
	Mean	28.0
	SD	3.95
	Median	27
	Range: min, max	18.0, 42.0
	95% CI	(27.6,28.4)
Height (cm)	N	358
	Mean	164.4
	SD	7.99
	Median	165
	Range: min, max	138.0, 187.0
	95% CI	(163.6, 165.3)
Weight (kg)	N	358
	Mean	75.6
	SD	11.20
	Median	75
	Range: min, max	49.0, 130.0
	95% CI	(74.5, 76.8)
Systolic blood pressure (mmHg)	N	358
	Mean	135.2
	SD	7.26
	Median	140
	Range: min, max	100.0, 144.0
	95% CI	(134.4, 136.0)
Diastolic blood pressure (mm Hg)	N	358
	Mean	86.3
	SD	8.56
	Median	85
	Range: min, max	60.0, 110.0
	95% CI	(85.4, 87.2)
Duration of use (months)	N	358
	Mean	7.1
	SD	3.98
	Median	7
	Range: min, max	1.0, 21.0
	95% CI	(6.7, 7.5)

Table 2 provides an overview of the prevalent risk factors for diabetes and its complications, while Table 3 presents details of comorbidities.The study population demonstrated a considerable burden of comorbidities, with hypertension being the most prevalent condition, followed by obesity and dyslipidaemia. Furthermore, the study identified several prevalent risk factors in the population, including a sedentary lifestyle, family history of diabetes, smoking, high salt intake, and emotional stress. Tobacco use and excess alcohol intake were also documented in a sizeable number of patients.

**Table 2:** Risk factors involved in overall population (N=358)

Parameter	Statistics	Overall (N=358)
Emotional Stress	Yes, n (%)	92 (25.7)
	No, n (%)	266 (74.3)
Excess alcohol intake	Yes, n (%)	37 (10.3)
	No, n (%)	321 (89.7)
Family history of diabetes	Yes, n (%)	170 (47.5)
	No, n (%)	188 (52.5)
Obesity	Yes, n (%)	247 (69.0)
	No, n (%)	111 (31.0)
Salt	Yes, n (%)	124 (34.6)
	No, n (%)	234 (65.4)
Sedentary lifestyle	Yes, n (%)	172 (48.0)
	No, n (%)	186 (52.0)
Smoking	Yes, n (%)	137 (38.3)
	No, n (%)	221 (61.7)
Tobacco	Yes, n (%)	45 (12.6)
	No, n (%)	313 (87.4)

**Table 3:** Comorbid conditions in overall population (N=358)

Parameter	Statistics	overall (N=358)
Coronary artery disease	Yes, n (%)	29 (8.1)
	No, n (%)	329 (91.9)
Dyslipidemia	Yes, n (%)	228 (63.7)
	No, n (%)	130 (36.3)
Erectile dysfunction	Yes, n (%)	11 (3.1)
	No, n (%)	347 (96.9)
Foot ulcer	Yes, n (%)	3 (0.8)
	No, n (%)	355 (99.2)
Hypertension	Yes, n (%)	293 (81.8)
	No, n (%)	65 (18.2)
Nonalcoholic fatty liver disease	Yes, n (%)	62 (17.3)
	No, n (%)	296 (82.7)
Nephropathy	Yes, n (%)	15 (4.2)
	No, n (%)	343 (95.8)
Neuropathy	Yes, n (%)	53 (14.8)
	No, n (%)	305 (85.2)
Retinopathy	Yes, n (%)	27 (7.5)
	No, n (%)	331 (92.5)
Transient ischemic stroke	Yes, n (%)	7 (2.0)
	No, n (%)	351 (98.0)
Other		
Chronic kidney disease (%)		2 (0.6)
Gangrene in scrotal area (%)		1 (0.3)
Insomnia (%)		4 (1.1)
Lower respiratory tract infection (%)		1 (0.3)
Migraine (%)		1 (0.3)
Post percutaneous transluminal coronary angioplasty (%)		1 (0.3)
Psoriasis (%)		1 (0.3)
Rheumatoid arthritis (%)		1 (0.3)

Efficacy outcomes

HbA1c: At baseline, the mean HbA1c level was 8.9 ± 0.87%. After three months (12 weeks) of treatment, the mean HbA1c level reduced to 7.2 ±0.78%. The mean change in HbA1c from the baseline to the third month was -1.7% (p <0.001).

FPG: Over the three-month period, there was a consistent reduction in FPG levels. At one month, the mean FPG level reduced form baseline with a mean change of -22.0 mg/dL. At two months, the mean FPG level further decreased, with a mean change of -39.5 mg/dL from baseline. Finally, at three months (12 weeks), the mean FPG level showed a mean change of -54.8 mg/dL from baseline (p<0.001).

PPG: At baseline, the mean PPG was 273.9 ±57.72 mg/dL. The mean change in PPG from baseline was -97.9 mg/dL in the third month (p <0.001).

**Figure 1:**
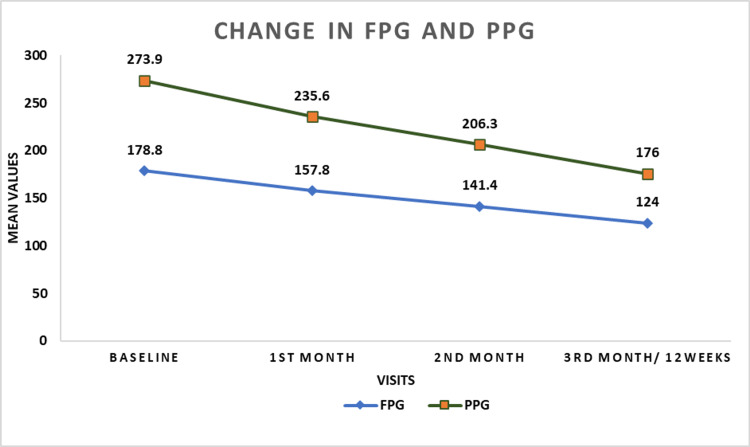
Change in FPG and PPG in overall population (N=358) FPG: Fasting plasma glucose; PPG: Postprandial plasma glucose

**Figure 2:**
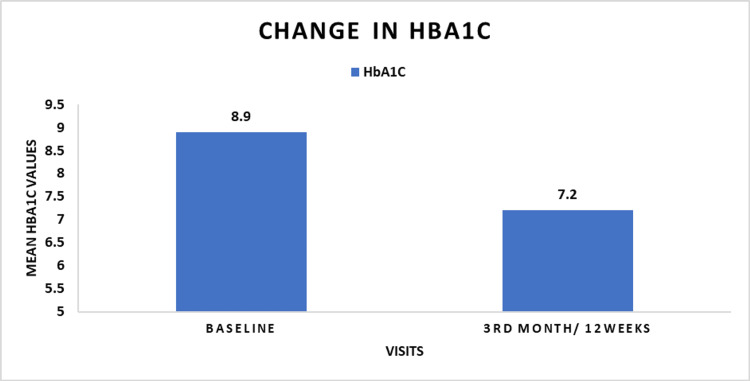
Change in HbA1c in overall population (N=358) HbA1c: Glycated hemoglobin

Table 4 describes the changes in HbA1c, FPG, and PPG. Figure 1 describes change in FPG and PPG level. Figure 2 describes change in HbA1c value.

**Table 4:** Change in HbA1c, FPG and PPG in overall population (N=358) HbA1c: Glycated hemoglobin; FPG: Fasting plasma glucose; PPG: Postprandial plasma glucose

Visit	Statistics	FPG	HbA1C	PPG
Baseline	n	358	358	358
	Mean	178.8	8.9	273.9
	SD	46.39	0.87	57.72
	Median	165	9	280
	Range: min, max	100.0, 332.0	6.6, 14.0	130.0, 478.0
	95% CI	(174.0, 183.6)	(8.8, 9.0)	(267.9, 279.9)
First month	n	306		305
	Mean	157.8		235.6
	SD	36.12		45.80
	Median	150		235
	Range: min, max	85.0, 280.0		135.0, 400.0
	95% CI	(153.8, 161.9)		(230.5, 240.8)
	n, Mean change (SD)	306, -22.0 (19.2)		305, -40.4 (28.2)
Second month	n	301		300
	Mean	141.4		206.3
	SD	28.24		38.76
	Median	140		200
	Range: min, max	80.0, 265.0		124.0, 350.0
	95% CI	(138.2, 144.6)		(201.9, 210.7)
	n, Mean change (SD)	301, -39.5 (28.5)		300, -72.2 (39.5)
Third month (12 weeks)	n	358	358	358
	Mean	124.0	7.2	176.0
	SD	22.54	0.78	33.58
	Median	125	7	176
	Range: min, max	70.0, 255.0	5.1, 11.0	100.0, 345.0
	95% CI	(121.6, 126.3)	(7.1, 7.3)	(172.5, 179.5)
	n, Mean change (SD)	358, -54.8 (36.3)	358, -1.7 (1.0)	358, -97.9 (49.0)
	p-value	<0.0001	<0.0001	<0.0001

 

Secondary outcomes

The safety outcomes of the FDC of dapagliflozin and sitagliptin therapy were evaluated in patients with T2DM. The reported adverse events were hypoglycemia, weight change, urinary tract infection, genitourinary tract infection, and change in blood pressure.

Weight change: Among the total study population of 358 patients, 333 (93.0%) experienced a change in weight while taking the FDC of dapagliflozin and sitagliptin. Out of these, 170 (47.5%) patients reported a weight loss of up to 2 kg, 134 (37.4%) patients experienced a weight loss of 2 to 4 kg, and 29 (8.1%) patients had a weight loss of more than 4 kg. Only 25 (7.0%) patients did not experience any weight change during the study period.

Hypoglycemia: Of the total 358 patients, 81 (22.6%) experienced hypoglycemia during the 12-week period. Among those who experienced hypoglycemia, only 2 (0.6%) suffered from severe hypoglycemic episode. The majority of patients did not experience any hypoglycemia events.

Urinary tract infection and genitourinary tract infection: Out of the 358 participants in the overall population, 43 (12.0%) experienced urinary tract infection or genitourinary tract infection events during the 12-week period. No other adverse events were reported in any of the participants.

Change in blood pressure: Before the treatment, the mean diastolic blood pressure was 93.1 ±12.02 mmHg, and the mean systolic blood pressure was 143.4 ±13.37 mmHg. After the treatment, the mean diastolic blood pressure decreased to 85.9 ±8.97 mmHg, and the mean systolic blood pressure decreased to 132.1 ±9.49 mmHg. The change in diastolic blood pressure was statistically significant with a mean change of 7.1 mmHg, p<0.001. Similarly, the change in systolic blood pressure was also statistically significant with a mean change of 11.3 mmHg, p<0.001.

Physicians' global evaluation of efficacy

Physicians' global evaluation of efficacy and tolerability has been depicted in Table 5. The efficacy of therapy FDC of dapagliflozin and sitagliptin was rated as excellent in 52.8% and very good in 32.1% of patients by physicians. The tolerability of the therapy was rated as as excellent in 46.4% and very good in 34.9% of patients by physicians.

**Table 5:** Physicians' evaluation in overall population (N=358)

Parameter	Statistics	Overall (N=358)
Physicians' global evaluation of efficacy	Average, n (%)	5 (1.4)
	Fair, n (%)	3 (0.8)
	Good, n (%)	46 (12.8)
	Very good, n (%)	115 (32.1)
	Excellent, n (%)	189 (52.8)
Physician global evaluation of tolerability	Average, n (%)	9 (2.5)
	Fair, n (%)	4 (1.1)
	Good, n (%)	54 (15.1)
	Very good, n (%)	125 (34.9)
	Excellent, n (%)	166 (46.4)

## Discussion

The use of FDCs of anti-diabetic drugs has gained popularity as an effective approach to managing multiple pathophysiological defects in T2DM with a single therapy, which can significantly improve treatment adherence [12]. In this retrospective study, we aimed to assess the efficacy and safety of a FDC of dapagliflozin and sitagliptin in the Indian population. To the best of our knowledge, this is the first real-world study to assess the effectiveness of this combination in the Indian population. The study revealed that this FDC is effective in improving glycaemic control, weight reduction, and blood pressure management in patients with T2DM. The study findings hold significant implications for healthcare professionals in India, given the high prevalence of diabetes in the country and the challenge it poses to diabetes management.

Our findings reveal that the use of this FDC effectively improves glycaemic control, as evidenced by a statistically significant decrease in HbA1c levels from baseline. These results align with previous studies that have demonstrated the efficacy of dapagliflozin and sitagliptin in enhancing glycaemic control among patients with T2DM. The observed reduction in HbA1c levels in our study (1.28%) is consistent with the range reported in earlier investigations (1.0% to 1.4%) [13-15]. In addition, most physicians rated the efficacy as very good or excellent. This finding holds implications for the clinical management of T2DM in India, where achieving and maintaining optimal glycaemic control is vital in preventing diabetes-related complications. Incorporating this combination therapy into individualized treatment plans can provide healthcare professionals with an additional treatment option. 

Additionally, our study demonstrates a reduction in body weight, which is in line with prior research indicating the weight-reducing effect of dapagliflozin and sitagliptin [16,17]. However, it should be noted that the magnitude of weight loss observed in our study was slightly lower than that reported in previous studies. This discrepancy could be attributed to the relatively shorter duration of treatment in our study (mean duration of 7.1 months) compared to the longer treatment duration in earlier investigations [16,17]. Furthermore, our study confirms the blood pressure-lowering effect of dapagliflozin and sitagliptin, as evidenced by a reduction in systolic blood pressure [18,19].

The adverse events were very few as observed in this study. Moreover, most physicians rated the tolerability as very good or excellent.

The real DAPSI findings have important practical implications for healthcare professionals in the management of patients with T2DM. The FDC therapy of dapagliflozin and sitagliptin was effective in reducing HbA1c levels, body weight, and blood pressure. This therapy can provide an alternative treatment option for patients with T2DM who have not achieved adequate glycaemic control with monotherapy or dual therapy.

Furthermore, our study reveals a high prevalence of comorbid conditions, particularly hypertension, dyslipidaemia, and obesity, among the study population. This underscores the importance of a comprehensive approach to managing T2DM in India. The FDC of dapagliflozin and sitagliptin not only targets glycaemic control but also offers potential benefits in terms of cardiovascular and renal protection, weight reduction, and blood pressure management [20].

This study also highlights the significant burden of risk factors and comorbidities among the study population. Healthcare professionals should be aware of these risk factors and comorbidities and take a holistic approach to the management of patients with T2DM. Interventions targeting modifiable risk factors, such as lifestyle modifications, can be incorporated into the treatment plan to improve outcomes in patients with T2DM.

The strengths of this retrospective study was a comprehensive analysis of the subject demographics, health status, and baseline clinical characteristics. Furthermore, the study was conducted in real-world settings, providing valuable insights into the effectiveness of the FDC in routine clinical practice. The study also had a mean duration of treatment of 7.1 months with the FDC, which allowed for the evaluation of safety and efficacy of the combination therapy. The study used multiple parameters to assess the safety and efficacy of the FDC, including the HbA1c levels, BMI, and blood pressure, which enhanced the robustness of the study results.

However, the current study has several limitations that must be acknowledged. The retrospective design limits the study's ability to control for potential confounding factors, leading to bias in the study results. Furthermore, the sample size is relatively small, limiting the study's power to detect significant differences in treatment outcomes. The study population also had a relatively short duration of treatment, limiting the generalizability of the findings to long-term treatment with the FDC of dapagliflozin and sitagliptin.

Future studies could build on the findings of this study and address some of its limitations. A randomized controlled trial could be conducted to assess the effectiveness of the FDC compared to other treatments. The study could also include a larger sample size, a more diverse population, and a longer follow-up duration to evaluate the long-term safety and efficacy of the combination therapy. Furthermore, future studies could focus on identifying the optimal dosing and duration of the FDC therapy and evaluating its cost-effectiveness. Finally, studies could assess the effects of the FDC on microvascular complications and quality of life measures in patients with T2DM.

## Conclusions

In conclusion, this real-world retrospective study set out to comprehensively evaluate the efficacy and safety of the FDC of dapagliflozin and sitagliptin in the Indian population, comprising 358 patients with T2DM. The study's findings showcase the significant impact of this combination therapy on glycaemic control, as evidenced by a significant reduction in HbA1c levels and consistent decreases in FPG levels over a three-month duration. Furthermore, the treatment demonstrated the added benefit of promoting weight loss. Notably, the occurrence of hypoglycemia was infrequent, occurring only in a minority of patients and primarily in mild or moderate forms. While a small proportion of participants reported urinary tract and genitourinary tract infections, these adverse events were manageable. These compelling results affirm the efficacy and general tolerability of the FDC of dapagliflozin and sitagliptin in the Indian population. However, it is essential to acknowledge the study's limitations, including its retrospective design and potential bias, necessitating further prospective studies to corroborate these findings and comprehensively evaluate the long-term safety and efficacy of this combination therapy. Overall, this study provides invaluable insights into the real-world effectiveness and safety of dapagliflozin and sitagliptin in managing T2DM among the Indian population.
